# miRandola: Extracellular Circulating MicroRNAs Database

**DOI:** 10.1371/journal.pone.0047786

**Published:** 2012-10-19

**Authors:** Francesco Russo, Sebastiano Di Bella, Giovanni Nigita, Valentina Macca, Alessandro Laganà, Rosalba Giugno, Alfredo Pulvirenti, Alfredo Ferro

**Affiliations:** 1 Department of Clinical and Molecular Biomedicine, University of Catania, Catania, Italy; 2 Department of Mathematics and Computer Science, University of Catania, Catania, Italy; 3 Department of Molecular Virology, Immunology and Human Genetics, Comprehensive Cancer Center, The Ohio State University, Columbus, Ohio, United States of America; National Institute of Health, United States of America

## Abstract

MicroRNAs are small noncoding RNAs that play an important role in the regulation of various biological processes through their interaction with cellular messenger RNAs. They are frequently dysregulated in cancer and have shown great potential as tissue-based markers for cancer classification and prognostication. microRNAs are also present in extracellular human body fluids such as serum, plasma, saliva, and urine. Most of circulating microRNAs are present in human plasma and serum cofractionate with the Argonaute2 (Ago2) protein. However, circulating microRNAs have been also found in membrane-bound vesicles such as exosomes. Since microRNAs circulate in the bloodstream in a highly stable, extracellular form, they may be used as blood-based biomarkers for cancer and other diseases. A knowledge base of extracellular circulating miRNAs is a fundamental tool for biomedical research. In this work, we present miRandola, a comprehensive manually curated classification of extracellular circulating miRNAs. miRandola is connected to miRò, the miRNA knowledge base, allowing users to infer the potential biological functions of circulating miRNAs and their connections with phenotypes. The miRandola database contains 2132 entries, with 581 unique mature miRNAs and 21 types of samples. miRNAs are classified into four categories, based on their extracellular form: miRNA-Ago2 (173 entries), miRNA-exosome (856 entries), miRNA-HDL (20 entries) and miRNA-circulating (1083 entries). miRandola is available online at: http://atlas.dmi.unict.it/mirandola/index.html.

## Introduction

MicroRNAs (miRNAs) are a class of approximately 22 nt long noncoding RNAs that mediate post-transcriptional gene regulation by repressing specific messenger RNA targets [1]. Like mRNAs, some miRNAs show restricted tissue distribution. For example, miR-122 is highly liver-specific, whereas miR-124 is preferentially expressed in neurological tissues [2], [3]. Changes in the spectrum of cellular miRNAs correlate with various physiopathological conditions including differentiation, inflammation, diabetes, and several types of cancers [4]–[10]. A significant amount of miRNAs has been found in extracellular human body fluids [11], [12]. Some circulating miRNAs in the blood have been successfully revealed as biomarkers for several diseases including cardiovascular diseases [13], cancer [11], [14], pediatric crohn disease [15], multiple sclerosis [16].

**Figure 1:**
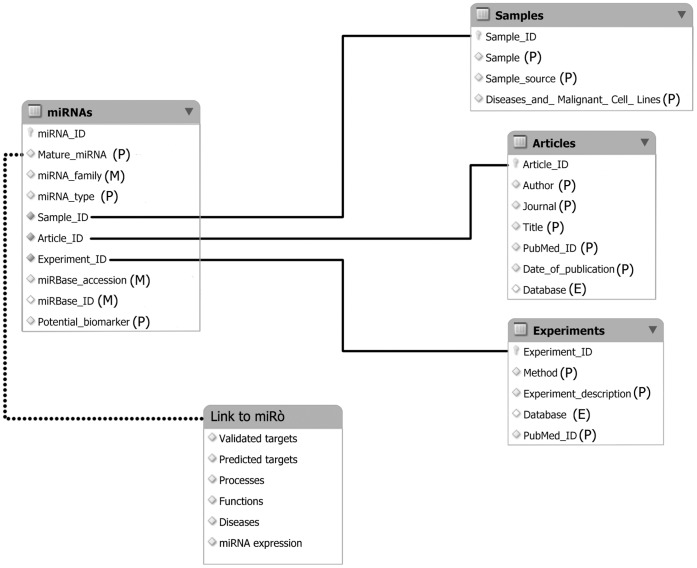
A schematic representation of architecture of database miRandola. (P): data taken from PubMed; (M): data taken from miRBase; (E): data taken from ExoCarta.

The dominant model for circulating miRNA stability is that miRNAs are released from cells in membrane-bound vesicles, which protect them from blood Rnase activity. Vesicles proposed as carriers of circulating miRNAs include exosomes, which are 50-nm to 90-nm vesicles arising from multivesicular bodies and released by exocytosis [17]. However, it has been reported that a significant portion of circulating miRNAs in human plasma and serum is associated with Argonaute2 (Ago2) [18], [19]. Ago2 is the effector component of the miRNA-induced silencing complex (miRISC) that directly binds miRNAs and mediates messenger RNA repression in cells [20], [21]. Although exosomal miRNAs have been hypothesized to be involved in intercellular communication [22], [23], most extracellular miRNAs might be by-products of dead cells that remain in extracellular space due to the high stability of the Ago2 protein and Ago2-miRNA complex [19]. These recent findings suggest that the analysis of miRNAs as biomarkers should include all kinds of circulating miRNAs found in biological fluids. Furthermore, despite the state of the art literature does not distinguish among the different forms of circulating miRNAs, we believe that a precise classification is needed. In this paper we present miRandola, a comprehensive database of extracellular/circulating miRNAs. miRNAs are classified into four categories, based on their extracellular form: miRNA-Ago2, miRNA-exosome, miRNA-HDL (High-density lipoprotein) and miRNA-circulating. The database provides users with a variety of information including the associated diseases, the samples, the methods used to isolate the miRNAs and the description of the experiment. Information about miRNA targets and their annotations are provided through links to miRò, the miRNA knowledge base [24]. miRò integrates data from different sources to allow the identification of associations among genes, processes, functions and diseases at the miRNA level through their predicted and validated targets (Figure 1).

miRandola is the first database that gathers and classifies data concerning kinds of extracellular miRNAs. It is manually curated and constantly updated by the authors, in order to include new data as soon as they are made available. miRandola allow users to contribute to the project through an online data submission form.

## Results and Discussion

### Data Sources and Web Interface

Data is manually collected from ExoCarta [25], a database of exosomal proteins, RNA and lipids and PubMed (http://www.ncbi.nlm.nih.gov/pubmed/) (Table S1). The aim of miRandola is to collect data concerning the miRNAs contained not only in exosomes but in all kind of circulating miRNAs functionally enriched with information such as their family, diseases, processes, functions, associated tissues, and their potential roles as biomarkers.

**Figure 2:**
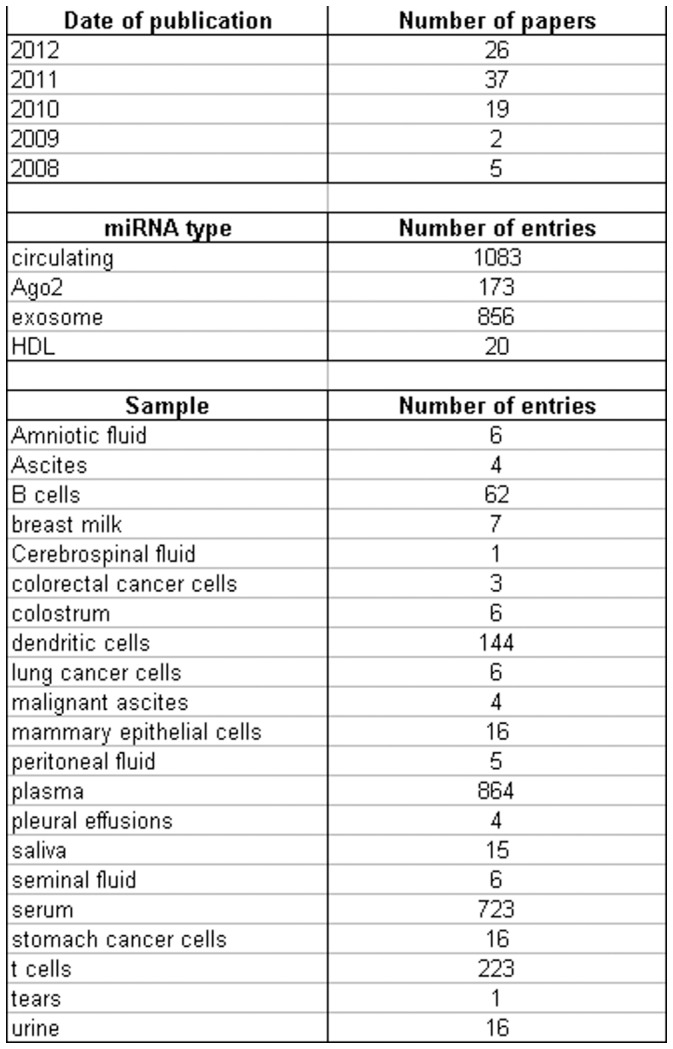
Statistics of important fields included in miRandola.

In particular, articles containing information on circulating miRNAs are collected by querying PubMed database using the keywords “microRNA”, “miRNA”, “extracellular” and “circulating”. Data is then manually extracted from the retrieved papers. General information about the miRNAs is obtained from miRBase [26].

**Figure 3:**
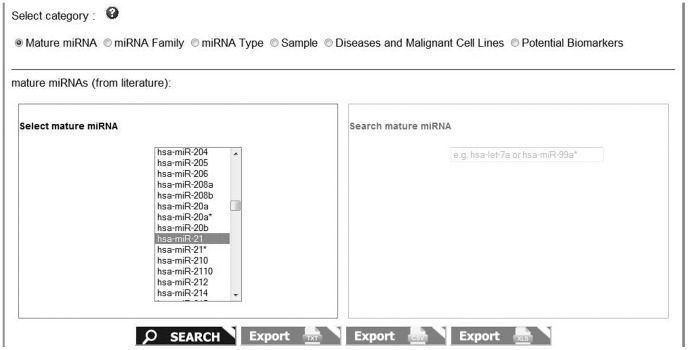
Keyword search.

**Figure 4:**
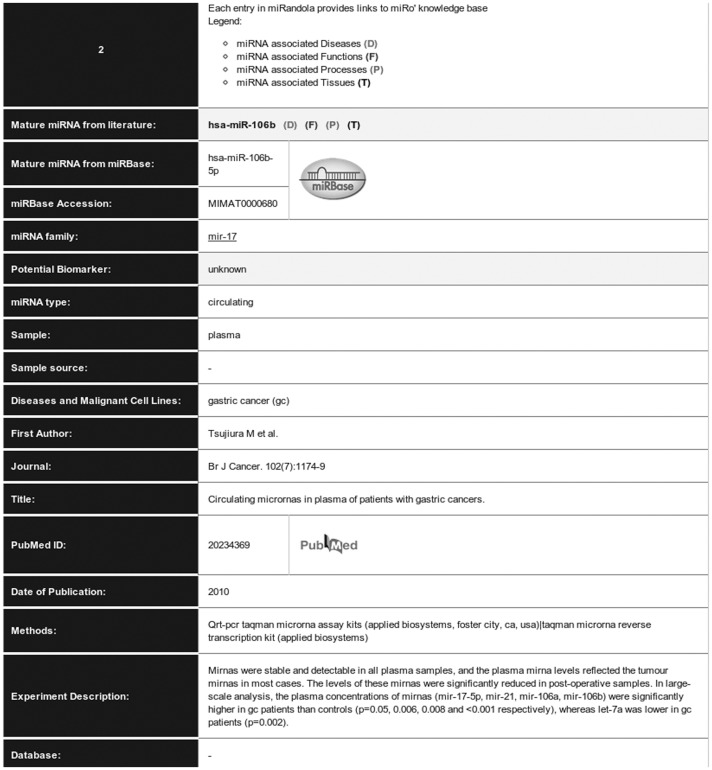
Keyword search results.

The miRandola database contains 2132 entries, with 581 unique mature miRNAs and 21 types of samples. miRNAs are classified into four categories, based on their extracellular form: miRNA-Ago2 (173 entries), miRNA-exosome (856 entries), miRNA-HDL (20 entries) and miRNA-circulating (1083 entries). The latter is used when authors do not distinguish between Ago2, exosome or HDL and constitutes the largest group. 864 and 723 entries derive from plasma and serum, respectively. To date, miRandola contains information gathered from 89 papers. The descriptive statistics of the database are shown in Figure 2.

**Figure 5:**
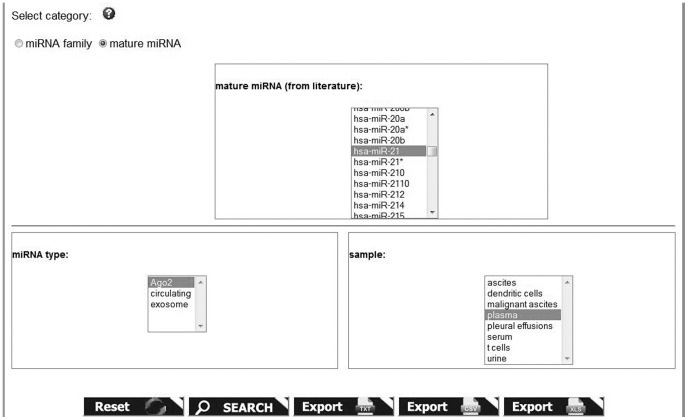
Advanced search.

All the data are collected and maintained up-to-date in a MySQL database running on an Apache server. The web interface has been implemented using PHP for the server side scripts and HTML and CSS for the client side coding. The database tutorial is available in the help section of the web site. miRandola has been tested using last version of Mozilla Firefox, Google Chrome, Safari and Internet Explorer.

### Utility Tools

For better data retrieval and analysis we have implemented various tools such as search, advanced search and browsing. These tools are described as follows.

### Keywords Search and Advanced Search

A simple text search and a browsing tool are provided for fast retrieval of data based on a single keyword. The database is searchable and browseable by Mature miRNA, miRNA Family, Sample, Diseases and Malignant Cell Lines, and Potential Biomarker Role (Figure 3). The results table includes data such as the mature miRNA name (e.g. hsa-miR-106b), the sample type (e.g. plasma), the related disease (e.g. Gastric Cancer), the circulating miRNA form (e.g. miRNA-circulating), information about the data source and external links to literature references (first author, journal, date of publication, title, PubMed ID) (Figure 4). The search results also include links to miRò, the miRNA knowledge base [24]. In particular, miRNAs are linked to the processes, functions, tissues and diseases.

The advanced search represents a more powerful way to query the database. Users can specify and combine different search terms in order to get more refined results. The screenshot in Figure 5 shows an example of advanced search page, in which we asked miRandola if miR-21 is found complexed with the Ago2 protein in plasma. The results of the queries are presented in tables and can be exported to various formats such as CSV, XLS and TXT.

### Circulating miRNAs Annotation

Although the function of circulating miRNAs is still largely unknown, there have been reported cases where endogenous miRNAs being transported by High-density lipoprotein (HDL) were delivered to recipient cells and contributed to the repression of their targets [27]. Moreover, exosomes seem to play an important role in the development of metastases, although the function of miRNA targeting sites that are distant from the primary organ is still unknown [28].

Therefore, in order to help formulating hypotheses on the function of secretory miRNAs, we connected miRandola to the miRò knowledge base [24], a web environment which provides users with miRNA functional annotations inferred through their validated and predicted targets.

In particular, each entry in miRandola provides links to the diseases, processes and functions in which the corresponding miRNA is involved and the tissues in which it is expressed.

### Online Data Submission

Another important feature that we implemented in miRandola allows a collaborative extension of the knowledge base. Users can give their contribution to the project by submitting new data about extracellular/circulating miRNA. The data, which can be uploaded through an online submission form, is added to the database and made available to the users after careful review performed by the miRandola staff. We believe this feature can help significantly to improve and extend the system, in the spirit of modern collaborative research projects [29].

### miRNA ID Converter

We have retrieved most of the information from articles published from 2008 to 2012. The majority of these papers referred to older versions of miRBase [26]. Moreover, the authors of miRBase recently reviewed their nomenclature system. These changes in miRNA IDs can generate misunderstanding and affect the identification and the retrieval of the desired data. For this reason, each entry in miRandola contains both the miRNA ID used in the source paper and the latest miRBase official ID. In addition, we have also included a useful online conversion tool providing, for each miRNA, the history of IDs from release 12 of miRBase to release 18.

## Analysis

A consistent number of recent studies demonstrated that exosomes derived from several cell lines may contain miRNAs [22], [23]. Other experiments revealed that >97% of extracellular miRNAs are exosome free [19]. Moreover, another study confirmed that a minority of plasma miRNAs are vesicle-associated, whereas potentially 90% of circulating miRNAs are present in a non-membrane-bound form consistent with a ribonucleoprotein complex [18].

Circulating miRNAs showed a strong discriminatory power in several diseases. miR-1 may be a novel biomarker for the diagnosis of acute myocardial infarction [30] and ST elevation myocardial infarction [31]. Vesicle-related miRNAs (let-7f and mir-30e-3p) in plasma of non-small cell lung cancer patients distinguished between two groups of patients for stage of disease and therefore possibility of surgery [32].

These recent findings suggest that it is very important to distinguish between the different kinds of circulating miRNAs, in order to (i) improve our knowledge on noninvasive biomarkers, (ii) infer possible biological functions of circulating miRNAs and (iii) their connection with the phenotypes.

### Future Perspectives

miRandola is the first online resource which gathers all the available data on circulating miRNAs in a unique environment. It represents a usufeul reference tool for anyone investigating the role of extracellular miRNAs as biomarkers as well as their physiological function and their involvement in pathologies. Current version of miRandola is focused on human circulating miRNAs. Future extensions will include information on different species and different circulating small RNA types. miRandola is constantly updated by the staff as soon as new data is available and the online submission system is a crucial feature which helps ensuring that the system is always up-to-date.

### Availability and Requirements

miRandola is available online at http://atlas.dmi.unict.it/mirandola/index.html.

## Supporting Information

Table S1 Papers in miRandola.
(PDF)
