# Supplementary material for: miRandola: Extracellular Circulating MicroRNAs Database
Source: PLoS One. 2012 Oct 19;7(10):e47786. doi: 10.1371/journal.pone.0047786 (PMC3477145; doi:10.1371/journal.pone.0047786)
Supplement: Table S1 — Papers in miRandola. (PDF) [file pone.0047786.s001.pdf]

miRandola is manually curated by extracting information from the following scientific results:

#	First Author	Journal	PubMed ID	Date of Publication
1	Goren Y et al.	Eur J Heart Fail. 14(2):147-54	22120965	2012
2	Hennessey PT et al.	PLoS ONE 7(2): e32307.	22389695	2012
3	Zhu HT et al.	Mol Biotechnol. 50(1):49-56.	21567136	2012
4	Tomimaru Y et al.	J Hepatol. 56(1):167-75.	21749846	2012
5	Wang LG et al.	Cancer Epidemiol. 36(1):e61-7.	22018950	2012
6	Liu H et al.	Cancer Lett. 316(2):196-203.	22169097	2012
7	Song J et al.	Dig Dis Sci. 57(4):897-904.	22198701	2012
8	Yu J et al.	J Cancer Res Clin Oncol. 138(4):671-4.	22237456	2012
9	Schwarzenbach H et al.	Breast Cancer Res Treat.	22350790	2012
10	Zhao Z et al.	Clin Chem.	22395025	2012
11	Tsai KW et al.	Genes Chromosomes Cancer. 51(4):394-401.	22420029	2012
12	Ding X et al.	Mol Med Report. 5(6):1428-32.	22427142	2012
13	Huang JJ et al.	Med Oncol.	22447484	2012
14	Yu S et al.	J Clin Endocrinol Metab.	22472564	2012
15	Chen ZH et al.	Prostate. doi: 10.1002/pros.22495.	22298030	2012
16	Bryant RJ et al.	Br J Cancer. 106(4):768-74.	22240788	2012
17	van Schooneveld E et al.	Breast Cancer Res. 14(1):R34.	22353773	2012
18	Siegel SR et al.	Mol Biol Rep. 39(5):6219-25.	22231906	2012
19	Wang B et al.	J Cancer Res Clin Oncol.	22638884	2012
20	Roth C et al.	PLoS One. 7(6):e38248.	22675530	2012
21	Long G et al.	Int J Biol Sci. 8(6):811-8.	22719221	2012
22	Gallo A et al.	PLoS One. 7(3):e30679.	22427800	2012
23	Yang C et al.	Int J Cancer. doi:10.1002/ijc.27657.	22674182	2012
24	Zahm AM et al.	J Pediatr Gastroenterol Nutr.	22732895	2012
25	Cookson VJ et al.	Cell Oncol (Dordr).	22821209	2012
26	Martino F et al.	PLoS One. 7(6): e38269.	22715378	2012
27	Rong H et al.	J Psychiatr Res. 45(1):92-5.	20546789	2011
28	Gui J et al.	Clin Sci (Lond). 120(5):183-93.	20815808	2011
29	Asaga S et al.	Clin Chem. 57(1):84-91	21036945	2011
30	Xu J et al.	Mol Carcinog. 50(2):136-42.	21229610	2011
31	Kanemaru H et al	J Dermatol Sci. 61(3):187-93.	21273047	2011
32	Wang G et al.	Lupus. 20(5):493-500.	21372198	2011
33	Arroyo JD et al.	PNAS 108(12):5003-8	21383194	2011
34	Roth C et al.	Mol Oncol. 5(3):281-91.	21398193	2011
35	Cheng H et al.	PLoS One.6(3):e17745.	21445232	2011
36	Moussay E et al.	PNAS 108(16):6573-8	21460253	2011
37	McDonald JS et al.	Clin Chem. 57(6):833-40	21487102	2011
38	Zahm AM et al.	J Pediatr Gastroenterol Nutr. 53(1):26-33.	21546856	2011
39	Turchinovich A et al.	Nucleic Acids Res. 39 (16): 7223-7233.	21609964	2011
40	Li S et al.	Circulation. 124(2):175-84.	21690488	2011
41	Duttagupta R et al.	PLoS ONE 6(6): e20769.	21698099	2011
42	Gidlöf O et al.	Cardiology. 118(4):217-26.	21701171	2011
43	Gonzales JC et al.	Clin Genitourin Cancer. 9(1):39-45.	21723797	2011
44	Cermelli S et al.	PLoS One.6(8):e23937	21886843	2011
45	Xiao J et al.	J Transl Med. 9:159.	21943159	2011
46	Wulfken LM et al.	PLoS One. 6(9):e25787.	21984948	2011
47	Mittelbrunn M et al.	Nat Commun. 2:282.	21505438	2011
48	Rupp AK et al.	Gynecol Oncol. 122(2):437-46.	21601258	2011
49	Bihrer V et al.	PLoS ONE 6(10): e26971.	22066022	2011
50	Silva J et al.	Eur Respir J. 37(3):617-23.	20595154	2011
51	Oyama R et al.	J Dermatol Sci. 61(3):209-11.	21277746	2011

**52**	Wei J et al.	Chin J Cancer. 30(6):407-14.	21627863	2011
**53**	Baggish AL et al.	J Physiol. 589(Pt 16):3983-94.	21690193	2011
**54**	Shen J et al.	BMC Cancer. 11:374.	21864403	2011
**55**	Wang Y et al.	Nat Nanotechnol. 6(10):668-74.	21892163	2011
**56**	De Rosa S et al.	Circulation. 124(18):1936-44.	21969012	2011
**57**	Cui L et al.	PLoS One. 6(11):e27071.	22087245	2011
**58**	Gunel T et al.	Genet Mol Res. 10(4).	22095477	2011
**59**	Tutarel O et al.	Int J Cardiol.	22188991	2011
**60**	Jung EJ et al.	Cancer.	22370716	2011
**61**	Yang Q et al.	Clin Chim Acta. 412(23-24):2167-73.	21840305	2011
**62**	Ge Q et al.	Clin Chim Acta. 412(21-22):1989-94.	21806976	2011
**63**	Vickers KC et al.	Nat Cell Biol. 13(4):423-33.	21423178	2011
**64**	Huang Z et al.	Int J Cancer. 127(1):118-26.	19876917	2010
**65**	Ai J et al.	Biochem Biophys Res Commun.391(1):73-7.	19896465	2010
**66**	Wang GK et al.	Eur Heart J. 31(6):659-66.	20159880	2010
**67**	Cheng Y et al.	Clin Sci (Lond). 119(2):87-95.	20218970	2010
**68**	Tsujiura M et al.	Br J Cancer. 102(7):1174-9	20234369	2010
**69**	Li A et al.	Cancer Res. 70(13):5226-37	20551052	2010
**70**	Fichtlscherer S et al.	Circ Res. 107(5):677-84.	20595655	2010
**71**	Miyachi M et al.	Biochem Biophys Res Commun. 400(1):89-93	20696132	2010
**72**	Weber JA et al.	Clin Chem. 56(11):1733-41.	20847327	2010
**73**	Redell JB et al.	J Neurotrauma. 27(12):2147-56.	20883153	2010
**74**	Corsten MF et al.	Circ Cardiovasc Genet. 3(6):499-506.	20921333	2010
**75**	Zhang Y et al.	Clin Chem. 56(12):1830-8.	20930130	2010
**76**	Roth C et al.	Breast Cancer Res. 12(6):R90.	21047409	2010
**77**	Guo HQ et al.	Dis Markers. 29(5):251-8.	21206010	2010
**78**	Ohshima K et al.	PLoS One.5(10):e13247.	20949044	2010
**79**	Ho AS et al.	Transl Oncol. 3(2):109-13.	20360935	2010
**80**	Zampetaki A et al.	Circ Res. 107(6):810-7.	20651284	2010
**81**	Pigati L et al.	PLoS One. 5(10):e13515.	20976003	2010
**82**	Hanke M et al.	Urol Oncol. 28(6):655-61.	19375957	2010
**83**	Zhu W et al.	BMC Res Notes. 2:89	19454029	2009
**84**	Rabinowits G et al.	Clin Lung Cancer. 10(1):42-6.	19289371	2009
**85**	Chim SS et al.	Clin Chem. 54(3):482-90	18218722	2008
**86**	Gilad S et al.	PLoS One. 3(9):e3148	18773077	2008
**87**	Mitchell PS et al.	PNAS 105(30):10513-8	18663219	2008
**88**	Taylor DD et al.	Gynecol Oncol.110(1):13-21.	18589210	2008
**89**	Hunter MP et al.	PLoS One. 3(11):e3694.	19002258	2008
